# The Stress-Regulated Transcription Factor CHOP Promotes Hepatic Inflammatory Gene Expression, Fibrosis, and Oncogenesis

**DOI:** 10.1371/journal.pgen.1003937

**Published:** 2013-12-19

**Authors:** Diane DeZwaan-McCabe, Jesse D. Riordan, Angela M. Arensdorf, Michael S. Icardi, Adam J. Dupuy, D. Thomas Rutkowski

**Affiliations:** 1 Department of Anatomy and Cell Biology, University of Iowa Carver College of Medicine, Iowa City, Iowa, United States of America; 2 Department of Pathology, University of Iowa Carver College of Medicine, Iowa City, Iowa, United States of America; 3 Department of Internal Medicine, University of Iowa Carver College of Medicine, Iowa City, Iowa, United States of America; University of Washington, United States of America

## Abstract

Viral hepatitis, obesity, and alcoholism all represent major risk factors for hepatocellular carcinoma (HCC). Although these conditions also lead to integrated stress response (ISR) or unfolded protein response (UPR) activation, the extent to which these stress pathways influence the pathogenesis of HCC has not been tested. Here we provide multiple lines of evidence demonstrating that the ISR-regulated transcription factor CHOP promotes liver cancer. We show that CHOP expression is up-regulated in liver tumors in human HCC and two mouse models thereof. *Chop*-null mice are resistant to chemical hepatocarcinogenesis, and these mice exhibit attenuation of both apoptosis and cellular proliferation. *Chop*-null mice are also resistant to fibrosis, which is a key risk factor for HCC. Global gene expression profiling suggests that deletion of CHOP reduces the levels of basal inflammatory signaling in the liver. Our results are consistent with a model whereby CHOP contributes to hepatic carcinogenesis by promoting inflammation, fibrosis, cell death, and compensatory proliferation. They implicate CHOP as a common contributing factor in the development of HCC in a variety of chronic liver diseases.

## Author Summary

Liver cancer is the third most common cause of cancer death worldwide. It is most commonly caused by viral hepatitis, alcoholism, or obesity, all of which activate cellular stress responses in the liver. However, the contribution of these responses to disease pathogenesis was unknown. We found that expression of the stress-regulated transcription factor CHOP—widely thought to be anti-oncogenic because of its cell death-promoting properties—was associated with both human liver cancer and two mouse models thereof. In response to challenge with a tumor-causing agent, mice lacking CHOP developed fewer tumors, exhibited less cell death, compensatory cellular proliferation, and liver scarring (fibrosis), and showed lower expression of immune and inflammatory genes. These findings establish CHOP as a biomarker for liver cancer and demonstrate its importance in promoting liver tumor formation. They raise the possibility that promotion of tumorigenesis by CHOP is a common feature of liver cancer caused by viral infection, alcoholism, and obesity.

## Introduction

HCC constitutes nearly 90 percent of all liver cancers in the United States, and liver cancer is the fifth most common cancer as well as the third most common cause of cancer related death worldwide [1], [2]. Risk factors for HCC include obesity, alcoholism, and viral hepatitis, due to the hepatic inflammation, fibrosis, and cirrhosis associated with these conditions [3]–[5]. In contrast to most other cancers, HCC prevalence has more than doubled in the U.S. over the past 20 years [2]. Stimuli that predispose to HCC show a striking commonality in their association with stress in the endoplasmic reticulum (ER), as well as signaling through the related unfolded protein response (UPR) and integrated stress response (ISR) pathways [6]–[10]. However, it is not known whether either the UPR or ISR contributes to HCC pathogenesis.

The ER participates in diverse physiological processes including membrane protein folding and trafficking, calcium storage, drug detoxification, lipid and sterol synthesis, and various steps of lipid and sugar catabolism. ER stress is caused by disruption of the organelle's protein folding capacity, and can be induced by varied physiological and pathological stimuli [6]. ER stress is sensed by the UPR, which is initiated by three ER-resident transmembrane proteins and which culminates in alterations to gene expression that improve ER function. One of these initiating proteins is the ER-resident kinase PERK, which when activated phosphorylates the translation initiation factor eIF2α to transiently inhibit protein synthesis and to stimulate gene expression via the eIF2α-regulated transcription factor ATF4 [7], [8]. eIF2α can also be phosphorylated by other kinases in response to diverse stresses (classically: viral infection, amino acid deprivation, or heme deficiency). This pathway, termed the integrated stress response (ISR), thus shares many transcriptional targets with the UPR [9]. IRE1α and ATF6α initiate the other two branches of the UPR, but are not thought to be activated by stimuli that do not cause ER stress; thus, the ISR is characterized by eIF2α-dependent signaling in the absence of IRE1α and ATF6α signaling.

The UPR and/or ISR are implicated in the pathogenesis of multiple types of tumors including breast, colon, prostate, brain and lung [10]–[15]. Evidence for UPR activation in human HCC tumors also exists [16]. UPR- and ISR-mediated apoptosis appears to require the ATF4-dependent transcription factor CHOP (C/EBP Homologous Protein) [17]. Thus CHOP induction has been proposed as a strategy for ameliorating cancer, and pharmacological ER stresses that induce CHOP can kill cancer cells, including hepatomas, *in vitro* [18], [19]. However, in at least one case CHOP appears to promote oncogenesis, when it is fused by genomic rearrangement with either the FUS/TLS protein or the EWS protein [20]–[22]. Although the common association of conditions that predispose to HCC with activation of the UPR and ISR points to a possible role for these pathways in HCC pathogenesis, their contribution to the development and progression of liver cancer has not been tested.

## Results

### CHOP Is Upregulated in a Genetic Mouse Model of HCC

To gain insight into the role of the UPR and ISR in HCC, we first sought evidence of UPR activation in liver tumors generated using a Sleeping Beauty (*SB*) transposon insertional mutagenesis system. In this approach, *SB* transposons containing promoter/enhancer and splice donor and acceptor elements are mobilized at random to induce insertional mutations. The system is activated when *SB* transgenic animals are crossed with SBase-expressing animals. Integrated transposons can alter the splicing or expression of nearby genes, leading to both gain- and loss-of-function mutations, and the positive selection of transposon-induced mutations drives tumorigenesis. This approach has the advantage that it ties altered physiology to a common genetic origin, and avoids the pleiotropy associated with drug-induced models of oncogenesis [23]. Mice with a mobilized T2/Onc3 transposon, which was optimized to promote oncogenesis in epithelial cells, produced many hepatocellular carcinomas and adenomas, among other malignancies of epithelial origin [24]. In liver tumors, the locus into which the transposon integrated most frequently was that of *Rtl1*, the molecular function of which has not yet been characterized [24], [25].

**Figure 1:**
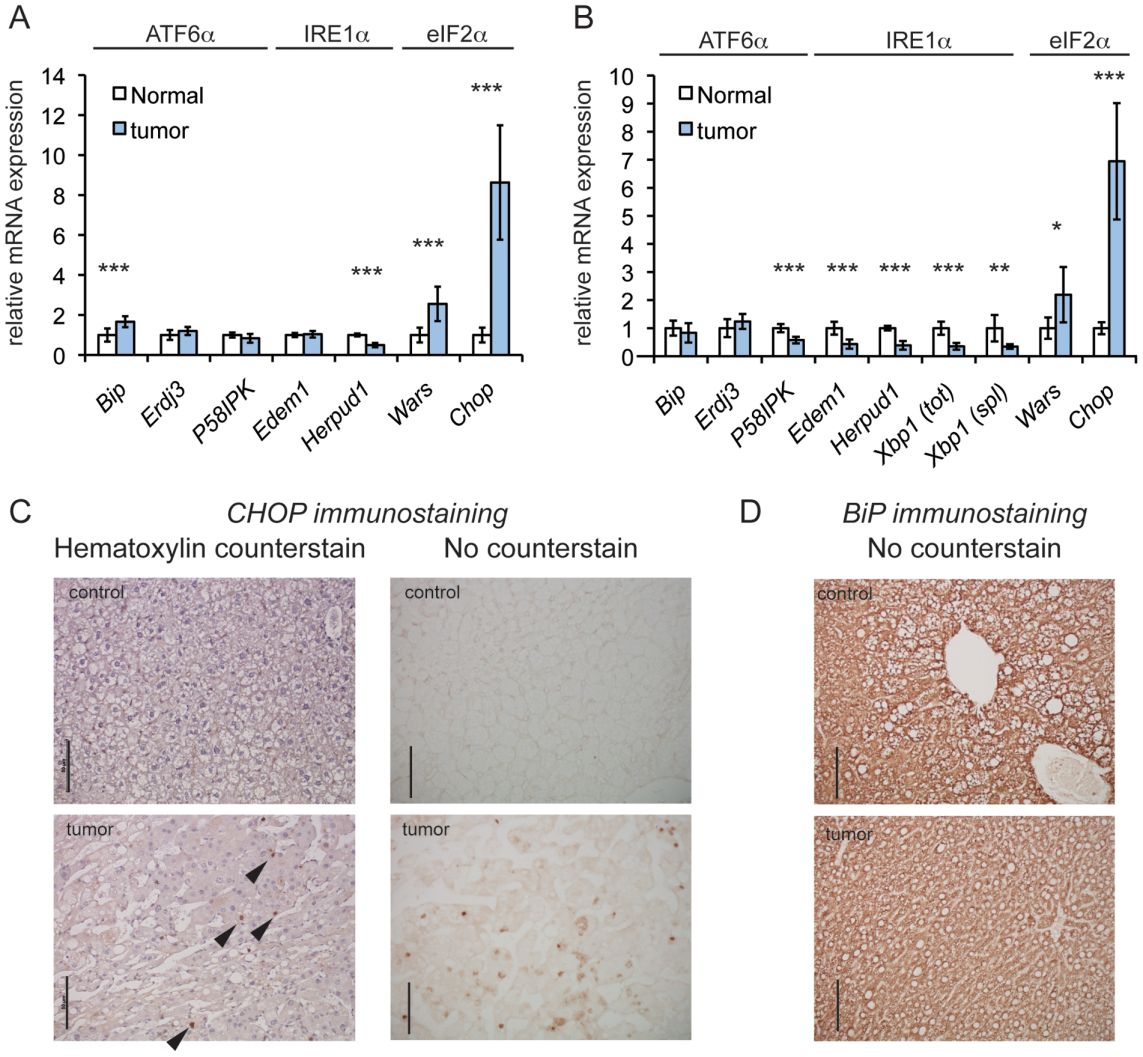
CHOP is upregulated in a genetic mouse model of HCC. (A) eIF2α-dependent genes are upregulated in tumors induced by *SB* mutagenesis. Relative expression of the indicated UPR genes in tumors versus normal mouse liver tissue, as quantified by transcriptome sequencing. Sequencing was performed on total RNA from mouse liver tumors generated by T2/Onc3 transposition into the *Rtl1* locus (n = 8) and age-matched normal liver tissue (n = 7). The branch of UPR signaling to which each gene is most responsive is indicated. Here and in (B), error bars represent means +/− S.D.M. Here and elsewhere: *, p<0.05; **, p<0.01; ***, p<0.001. (B) qRT-PCR of *SB*-induced tumors confirms upregulation of *Chop*. Relative expression of the indicated genes quantitated by qRT-PCR from tumors arising from T2/Onc3 *Rtl1* locus integrants (n = 7), compared to expression in normal tissue (n = 5). Relative expression of total (tot) and spliced (spl) *Xbp1* mRNA is also shown. mRNA levels were normalized against average expression of 2 housekeeping genes (*Btf3* and *Ppia*). (C and D) CHOP and BiP expression, respectively, in tumors from *Rtl1* locus integrants and normal liver tissue from age-matched control animals using IHC. Also shown for CHOP staining are hematoxylin-counterstained samples, with exemplar CHOP-positive nuclei indicated by arrowheads. All scale bars throughout this work represent 50 µm unless indicated otherwise. The same anti-mouse immunoglobulin secondary antibody was used for both CHOP and BiP immunostains.

The expression of UPR-regulated mRNAs in *Rtl1*-integrant tumors versus normal liver tissue was assessed as part of whole-transcriptome sequencing of total mRNA [25]. (Only *Rtl1* integrants were used because these made up the vast majority of SB-induced tumors, and the number of samples from non-*Rtl1* integrants was insufficient to allow for meaningful quantitative comparison.) This analysis revealed enrichment of the ISR-regulated genes *Chop* and *Wars*, as well as multiple additional ISR target genes (Figures 1A and S1) [7], [9], [26], [27], but little or no upregulation of ER chaperones and ER-associated degradation factors, which are more prominently regulated by the PERK-independent IRE1α and ATF6α pathways of the UPR [28], [29] (Figure 1A). This pattern of regulation was confirmed by quantitative RT-PCR (qRT-PCR) in independent tumor samples and normal liver controls (Figures 1B and S1). There was also no enrichment in tumors of the spliced form of *Xbp1* mRNA, which is produced when the IRE1α pathway of the UPR is activated and which serves as a general sentinel for UPR (but not ISR) activation [8] (Figure 1B). In immunohistochemical analysis using an antibody that we confirmed was specific for CHOP (Figure S2), tumor samples consistently displayed scattered CHOP-positive nuclei. CHOP immunostaining was observed in the two non-*Rtl1* tumors that were examined as well (unpublished data). In contrast, normal liver exhibited essentially no CHOP staining (Figure 1C). Consistent with our transcriptome sequencing and qRT-PCR results, the tumor samples did not differ from normal liver with respect to expression of the abundant ER chaperone BiP (Figure 1D). Taken together, these results suggest that HCC is characterized at the level of gene expression by activation of an ISR and upregulation of CHOP, but not by activation of a canonical UPR.

### CHOP Promotes Oncogenic Transformation *In Vivo*

**Figure 2:**
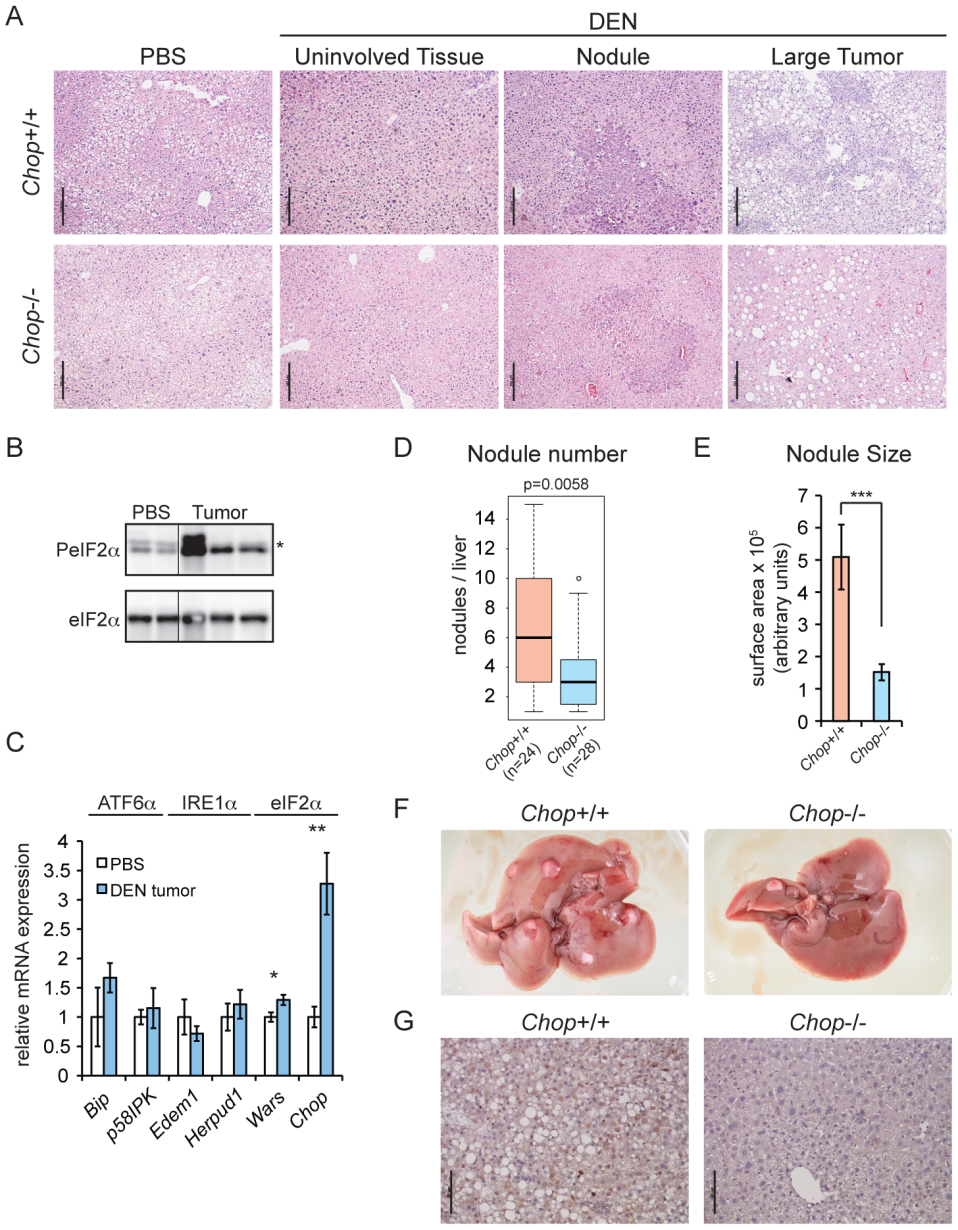
*Chop*−/− animals are resistant to DEN-induced tumorigenesis. (A) A single injection of DEN induces liver tumors in wild-type and *Chop*−/− mice. 15 d.o. wild-type (n = 24) and *Chop*−/− (n = 28) male mice were injected i.p. with 25 mg/kg DEN or PBS (n = 3 per genotype) and aged to 9 months. Liver sections were analyzed by hematoxylin and eosin staining. Histology of representative uninvolved tissue (i.e., tumor-free), nodules, and large tumors from both genotypes is shown. Scale bar = 100 µm. (B) DEN treatment induces eIF2α phosphorylation. Total and phosphorylated eIF2α were detected by immunoblot in normal liver and tumor tissue from wild-type animals. Each lane represents a separate animal. Hairline indicates electronic splicing of non-contiguous lanes, which were taken from the same exposure and processed identically. * represents a nonspecific band. (C) eIF2α target genes are upregulated in tumors from DEN-treated mice. The indicated mRNAs were quantitated by qRT-PCR as in Figure 1 from large tumors of DEN-injected wild-type animals (n = 3), compared against normal liver tissue from PBS-injected wild-type animals (n = 3). Error bars represent means +/− S.D.M. (D–F) Fewer and smaller tumors in *Chop*−/− mice. (D) Surface nodules were counted from the animals described in (A) above, blinded to genotype. P-value was calculated by Asymptotic Wilcoxon Rank Sum Test. (E) Smaller tumors in *Chop*−/− mice. Average cross-sectional area of nodules from H&E-stained liver slices of animals from (A) was determined using Image J, and is shown here +/− S.E.M. p-value was calculated by two-tailed student's t-test. (F) Representative liver photos derived from either wild-type or *Chop*−/− mice from (A) are shown. CHOP immunostaining was observed in all tumors or nodules from wild-type animals for which it was examined. PBS-injected animals of both genotypes were tumor-free (unpublished data) (G) CHOP induced by DEN is detected by immunohistochemistry. CHOP expression in liver sections derived from either wild-type or *Chop*−/− mice from (A) is shown by IHC, with a hematoxylin counterstain.

We next set out to determine whether CHOP promotes tumorigenesis *in vivo*. To test this hypothesis, 15 day-old male *C57BL/6J* and extensively (>10 generations) backcrossed *Chop*−/− mice, which have a constitutive deletion of the majority of the CHOP open reading frame [30], were given a single intraperitoneal injection of the liver carcinogen diethylnitrosamine (DEN), and then aged to 9 months. The DEN model was used for these experiments because, unlike the *SB* model, it is accompanied by hepatic inflammation and fibrosis, and thus more closely mimics the events associated with human HCC [31]. At 9 months, DEN treatment produces mostly basophilic foci and hepatocellular adenomas, and gives rise to carcinomas at later time points [32]. Animals of both genotypes formed multiple liver nodules and, occasionally, large tumors. As expected, nodules and tumors were characterized to varying degrees by hyperproliferation, pleiomorphism, vacuolization, and loss of cellular architecture characteristic of foci of cellular alteration, basophilic foci, or hepatocellular adenomas (Figure 2A). Tumors from DEN-treated mice showed elevated eIF2α phosphorylation (Figure 2B) and upregulation of *Wars* and *Chop* mRNA (Figure 2C), consistent with the idea that hepatic oncogenesis is associated with an activated ISR and upregulation of *Chop*.

We found that *Chop*−/− mice were significantly protected from DEN-induced tumorigenesis; the median number of liver surface nodules per animal was reduced by 50 percent compared to that in their wild-type counterparts, and nodules in *Chop*−/− mice had on average approximately one-third the cross-sectional surface area of nodules in wild-type animals (equivalent to approximately a 6-fold difference in nodule volume) (Figures 2D–F). The liver weight-to-body weight ratio was not significantly different between the two genotypes (unpublished data). Consistent with our findings for *SB*-induced tumors, CHOP-positive nuclei were seen by IHC in DEN-treated wild-type animals (Figure 2G). Thus, CHOP upregulation is common to both genetic and pharmacological models of HCC.

### CHOP Deletion Reduces Apoptosis and Proliferation

**Figure 3:**
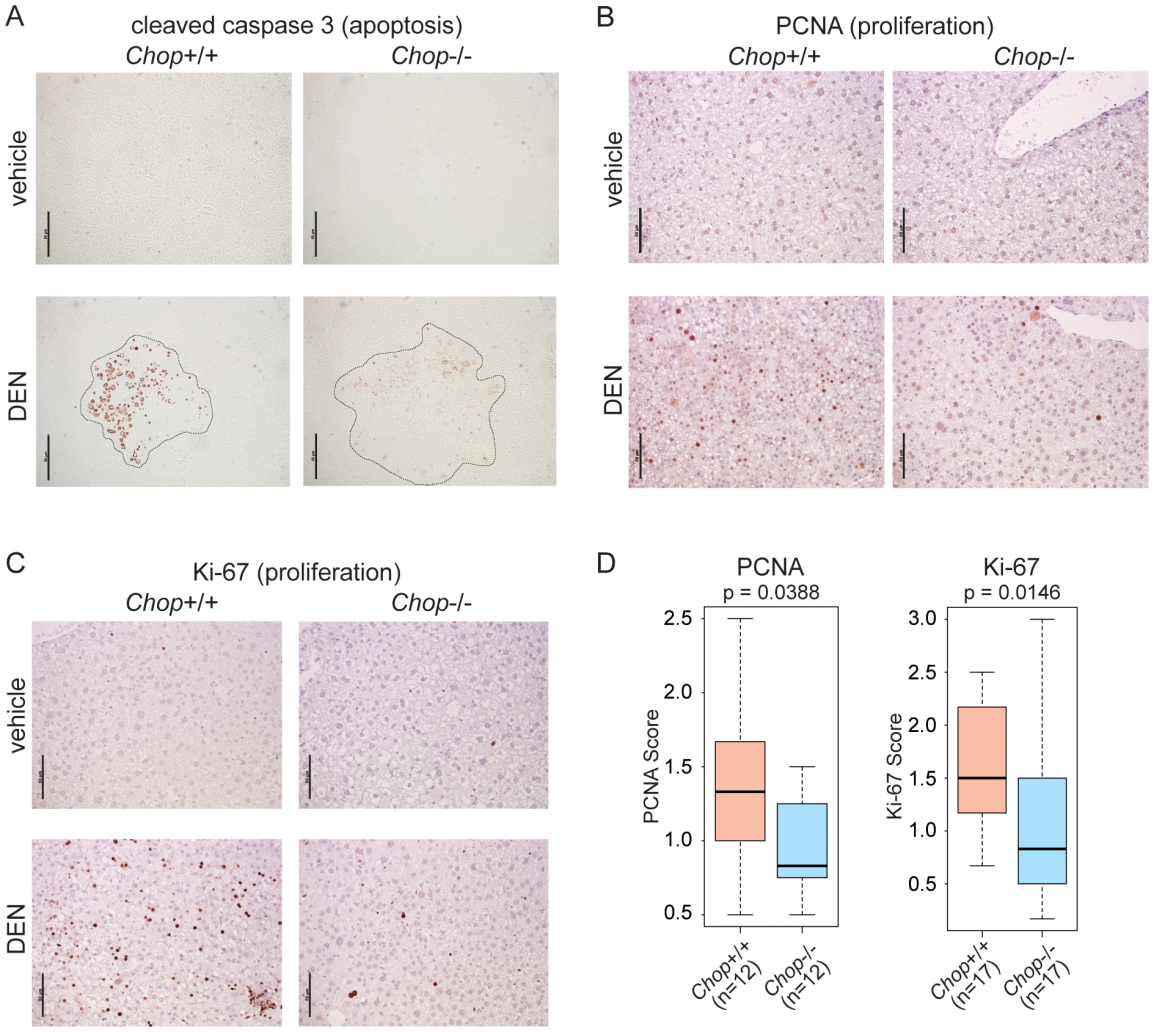
CHOP promotes hepatocellular apoptosis and proliferation. (A) *Chop*−/− mice show reduced cell death upon DEN challenge. Apoptosis in vehicle- or DEN-treated livers of wild-type or *Chop*−/− mice was detected by IHC staining for the cleaved form of Caspase-3. Liver nodule borders are indicated by a dashed outline. Hematoxylin counterstain is not shown because it interfered with Caspase-3 staining. In (A–C), representative images are shown. (B–D) Reduced proliferation in *Chop*−/− DEN-challenged animals. Hepatocellular proliferation was detected by IHC staining for PCNA (B) or Ki-67 (C). (D) The extent of PCNA and Ki-67 staining was quantitated blindly as described in the Materials and Methods. P-values were determined by Asymptotic Wilcoxon Rank Sum Test.

The strong association of CHOP with apoptosis in both cells and animal models of various diseases could have suggested an anti-oncogenic function for this protein. However, we hypothesized that CHOP might instead promote HCC by facilitating the compensatory proliferation that accompanies hepatocyte cell death and that is thought to characterize HCC [33]. This hypothesis predicted that in DEN-treated *Chop*−/− animals, the expression of *both* cell death and proliferative markers would be lower than in wild-type animals. Indeed, while staining for the active form of Caspase-3 revealed foci of extensive apoptosis primarily (though not exclusively) associated with nodules in wild-type mice, apoptosis was dramatically reduced in *Chop*−/− animals (Figure 3A). Proliferation, which was associated with both nodular and non-nodular areas in wild-type animals, was also attenuated in *Chop*−/− mice, as seen by staining for the proliferative markers PCNA and Ki-67 (Figure 3B and 3C). Blinded scoring revealed that the reduction of both markers in *Chop*−/− livers was significant (Figure 3D). These results show that CHOP promotes hepatocellular proliferation despite—or perhaps because of—its apoptotic function.

### CHOP Promotes Fibrosis

**Figure 4:**
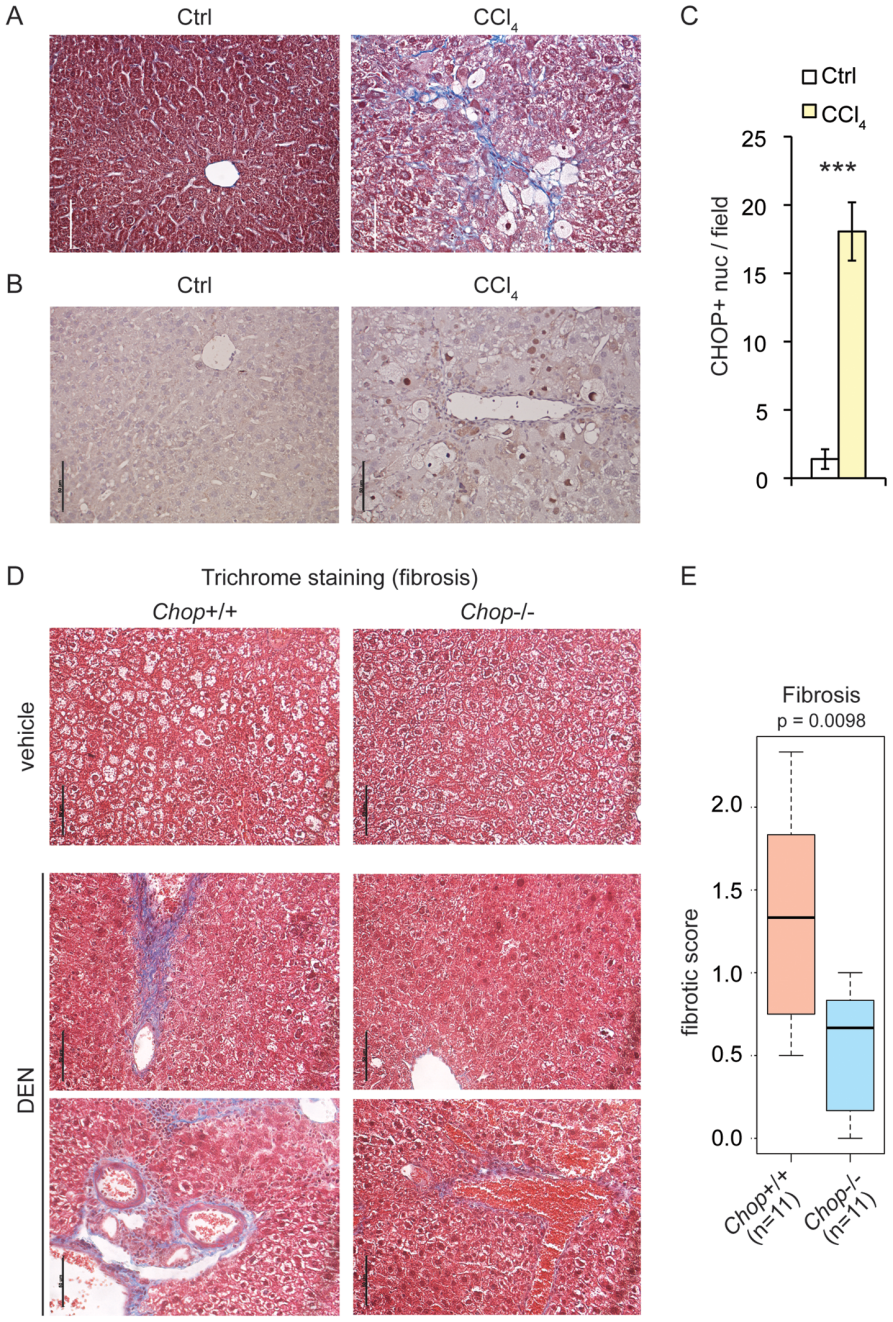
CHOP is induced by fibrotic insult and promotes fibrosis. (A) CCl_4_ injection promotes collagen deposition. Masson's trichrome staining was used to assess collagen deposition (blue) in formalin-fixed paraffin-embedded liver sections of mice injected i.p. with 10% CCl_4_ twice a week for 12 weeks, or with mineral oil as a control. (B–C) CHOP expression is associated with CCl_4_-challenged livers. (B) CHOP was detected by IHC in mouse livers from (A) and samples were counterstained with hematoxylin as in Figure 1C. Representative data are shown. (C) The number of CHOP-positive nuclei per microscopic field from (B) was quantitated in multiple fields from 3 mice per group, and is expressed here as mean +/− S.E.M. per field. (D–E) *Chop* deletion attenuates DEN-induced fibrosis. Mild-to-moderate fibrosis is seen in wild-type but not *Chop*−/− livers by trichrome staining after DEN treatment as in Figure 2. Sections from two separate DEN-treated animals of each genotype are shown. (E) A METAVIR score was blindly determined from trichrome stains as described in the Materials and Methods, and the p-value was calculated by Asymptotic Wilcoxon Rank Sum Test. 0 = no fibrosis; 1 = portal fibrosis lacking septa; 2 = portal fibrosis with some septa; 3 = abundant septa; 4 = cirrhosis.

The progression of alcoholic or non-alcoholic fatty liver disease to fibrosis sharply increases the risk of developing HCC [34]. A study of hepatic fibrosis in the bile duct ligation model of cholestatic liver injury revealed that this condition is promoted by CHOP [35]. More recently, however, CHOP was shown to protect animals from fibrosis induced by dietary steatohepatitis [36]. We found that chronic challenge of wild-type mice with the hepatotoxin carbon tetrachloride (CCl_4_) induced fibrotic deposition, as revealed by blue collagen fibers in Masson's trichrome staining (Figure 4A). CHOP immunostaining was evident in histological sections of these livers but not controls (Figure 4B, C), demonstrating that CHOP expression occurs not only in tumors and nodules, but also in association with a key upstream predisposing event (fibrosis).

Tumorigenesis following DEN treatment is accompanied by inflammation and hepatocyte injury [37], [38]. Repeated administration of DEN or the related DMN also leads to progressive fibrosis upon intoxicant cessation [31], [39]–[42]. Trichrome staining revealed that even a single post-natal DEN treatment led to mild-to-moderate fibrotic deposition in 9-month-old wild-type mice (Figure 4D). However, significantly less deposition was observed in *Chop*−/− mice (Figure 4D, E). These results demonstrate that CHOP expression is associated not only with tumors, but also with an agent that causes hepatocyte injury, and place CHOP upstream of the activation of fibrosis.

### CHOP Deletion Alters the Expression of Immune and Inflammatory Genes

**Figure 5:**
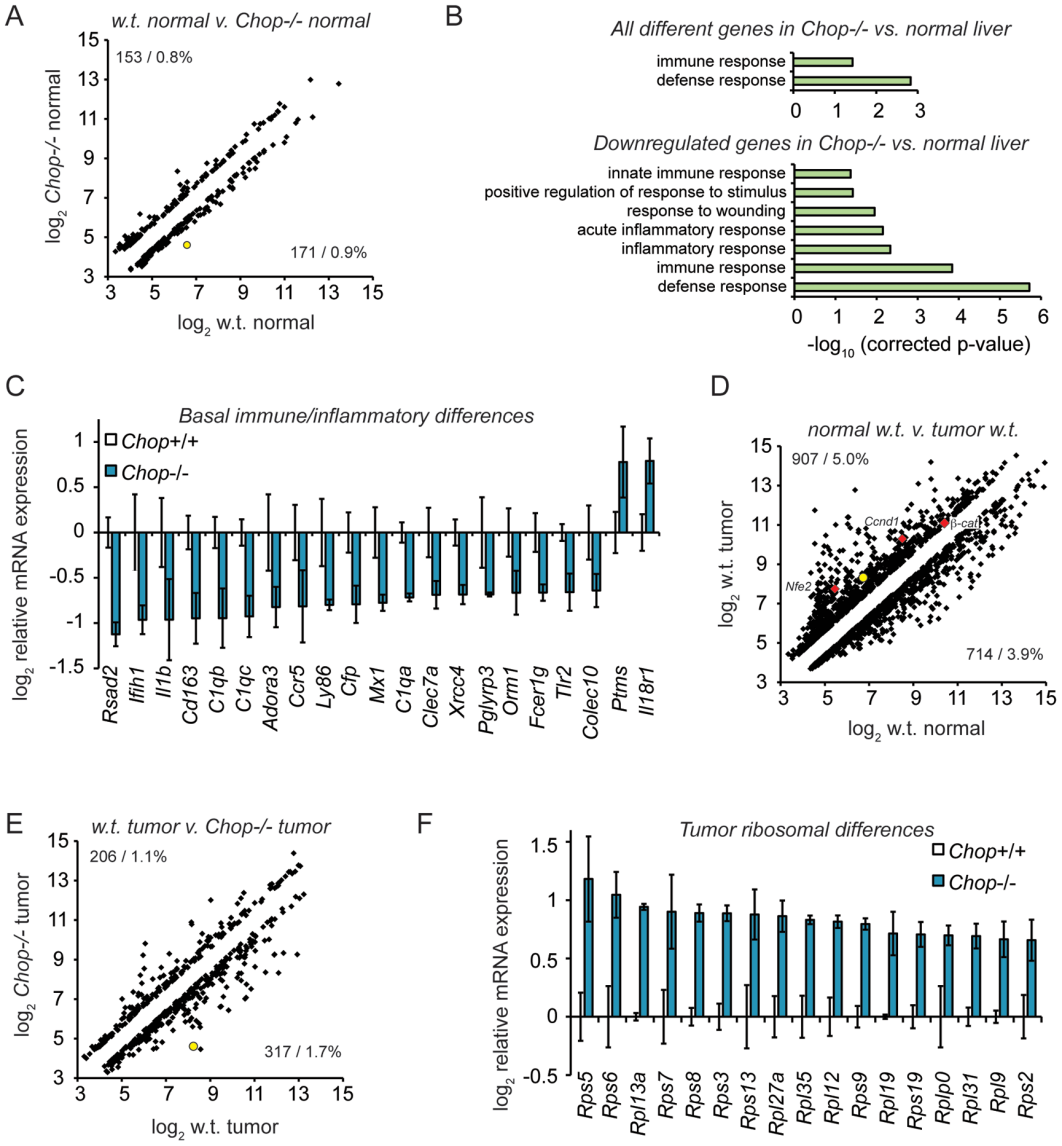
Microarray profiling reveals CHOP-dependent differences in inflammation and ribosomal biogenesis genes. (A) Basal expression differences in *Chop*−/− mice. 18,138 significantly expressed genes were analyzed using Illumina MouseRef-8 v2.0 BeadChips, with total RNA from 9 month old mouse liver homogenate as input. The number and percentage of genes upregulated (upper left) and downregulated (lower right) in wild-type versus *Chop*−/− tissue is shown, showing only genes differing by 1.5-fold or more, p<0.05. Expression is given on a log_2_ scale. In (A), (D), and (E), the position of *Chop* is shown as a yellow-filled circle. (B–C) Suppressed basal expression of genes involved in immune and inflammatory pathways in *Chop*−/− mice. (B, top) GO pathways enriched among all regulated genes in PBS-treated wild-type versus *Chop*−/− animals were determined using DAVID, with all pathways that were significantly enriched after Bonferroni correction shown. (B, bottom) Same as (top), but considering only genes downregulated in *Chop*−/− animals. (C) Average array-determined expression of genotype-dependent immune- and inflammation-related genes from PBS-treated animals is shown on a log_2_ scale +/− S.D.M. All genes shown are differentially expressed >1.5-fold (i.e., >0.585 on the y-axis), p<0.05. (D) DEN treatment alters gene expression in wild-type mice. Scatter plot of all genes differentially expressed (>1.5-fold; p<0.05) in tumors from 9 month-old DEN-treated wild-type animals versus normal liver tissue in age-matched PBS-treated wild-type animals. The positions of *Nfe2*, *Cyclin D1* (*Ccnd1*), and *β-catenin* are indicated by red diamonds. (E–F) Uniform upregulation of genes involved in ribosome biogenesis in tumors from *Chop*−/− mice. (E) Scatter plot of gene expression from tumor tissue of DEN-treated wild-type animals compared to *Chop*−/− animals is presented, showing all genes differing by >1.5-fold, p<0.05. (F) Average expression of all ribosomal subunit genes that were significantly different (p<0.05, fold-change >1.5) in *Chop*−/− versus wild-type large tumors is shown on a log_2_ scale +/− S.D.M. as in (C).

We next asked whether gene expression in *Chop*−/− mice differed from wild-type mice *independent of an inciting stimulus* in a way that could account for the greater susceptibility of wild-type mice to fibrosis and oncogenesis. To do this, total RNA was prepared from liver homogenates of 9-month-old wild-type and *Chop*−/− mice, and gene expression was profiled by microarray. A small group of genes (less than 2 percent of those represented on the array) were differentially expressed (>1.5-fold, p<0.05) in normal liver tissue from *Chop*−/− mice compared to wild-type animals (Figure 5A). Gene ontology (GO) pathway analysis of these genes revealed that this population was significantly enriched for mediators of innate immunity (Figure 5B and Table S1). The vast majority of these were expressed at lower levels in *Chop*−/− animals than in wild-type, and when only downregulated genes were considered in the pathway analysis, the significance of the immune and inflammatory GO pathway enrichments was strengthened (Figure 5B, C). Similar expression differences were seen in the livers of wild-type versus *Chop*−/− DEN-treated animals, although, because those samples included both nodular and non-tumorous tissue, gene expression was more variable in general (Figure S3). The expression of several immune and inflammatory genes was confirmed by qRT-PCR (unpublished data). These results are consistent with the known role of inflammation in promoting fibrosis and HCC [43], in that the protected *Chop*−/− animals show evidence for suppressed pro-inflammatory gene expression even in the absence of an overt hepatotoxic challenge. These differences were not seen in young (∼8 week-old) mice, suggesting that they are more likely to reflect long-term indirect effects of CHOP function than to identify direct transcriptional targets of CHOP.

We next compared gene expression in tumors derived from DEN-treated wild-type or *Chop*−/− mice, to determine whether CHOP influences not only the likelihood of tumor formation, but also gene expression in tumors once formed. As expected, tumors in DEN-treated wild-type mice showed many alterations to gene expression compared to vehicle-treated animals (>1.5-fold, p<0.05). These alterations included genes previously implicated in HCC that were upregulated in tumors including *Cyclin D1*, *β-catenin*, and *Nfe2* (Figure 5D).

Approximately 3 percent of genes were differentially expressed in *Chop*−/− tumors compared to normal tumors (>1.5-fold, p<0.05) (Figure 5E). Pathway analysis of CHOP-dependent differences in gene expression within tumors did not reveal significant enrichment of any pathway. However, when only genes that were *upregulated* in *Chop*−/− tumors were subjected to pathway analysis, the protein translation pathway (and only this pathway) was enriched to an extent that was highly significant (p∼2.6×10^−6^ after Bonferroni correction). Indeed, every gene without exception encoding a ribosomal protein that differed significantly with respect to tumor expression was expressed at higher levels in *Chop*−/− tumors than in wild-type tumors (Figure 5F). These results suggest that CHOP influences ribosomal biogenesis in tumors, although the effect is likely indirect, because none of these genes contains a consensus CHOP-binding site or is influenced by transient CHOP overexpression [44]. Whether these alterations result in enhanced ribosomal biogenesis and protein synthesis in *Chop*−/− tumors has yet to be determined.

### CHOP Expression Is Associated with Human HCC

**Figure 6:**
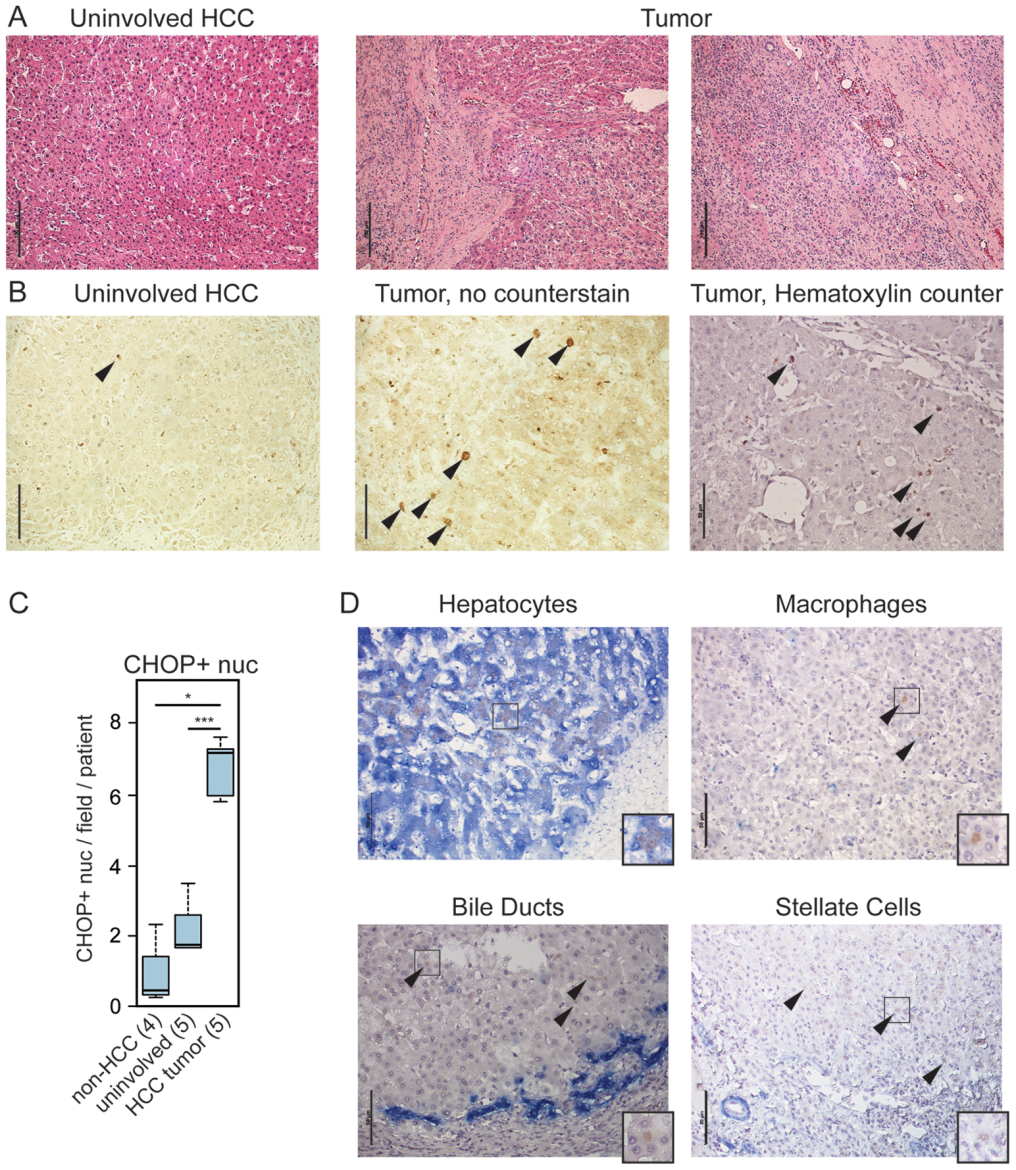
CHOP is expressed in human HCC tumors. (A) Histology of tumor-proximal liver (uninvolved HCC) and tumor (two regions) tissue from the same patient. H&E images are representative of 28 tumor samples and 5 uninvolved matched controls. For (A) only, scale bar = 100 µm (B–C) CHOP immunostaining is associated with human HCC but not uninvolved regions. (B) Samples from uninvolved tumor-proximal tissue and HCC tumors were probed for CHOP expression by immunohistochemistry. CHOP-positive nuclei are indicated by arrowheads. Cellular and nuclear architecture in some HCC samples was distorted as seen in the non-counterstained image, accounting for the large nuclei. Scale bar = 50 µm. (C) The average number of CHOP-positive nuclei per microscopic field per patient was quantitated blindly from 5 HCC tumors and their uninvolved matched controls, as well as from 4 non-HCC specimens, and these were aggregated by Boxplot. P-values were calculated by Asymptotic Wilcoxon Rank Sum Test. (D) HCC-associated CHOP is expressed in hepatocytes. Expression of CHOP (brown) and cell type-specific markers (blue) for hepatocytes (cytokeratin-18), macrophages (CD68), bile ducts (cytokeratin-7), or activated stellate cells (smooth muscle actin) is shown by immunohistochemistry, with a hematoxylin counterstain applied. Representative data are shown. Note that only hepatocytes show CHOP-positive nuclei; arrowheads are used to show CHOP-positive cells in other samples. Scale bar = 50 µm. Insets show 25 µm×25 µm regions with distinct CHOP-positive nuclei.

To determine the relevance of our findings to human disease, we asked whether CHOP expression was associated with human HCC. We examined archival HCC tumor tissue as well as unaffected liver regions from HCC patients (“uninvolved”) (Figure 6A) and non-HCC liver specimens. We first confirmed that our antibody detected human CHOP specifically (Figure S4). Using this antibody on human liver tissue we found extensive CHOP staining in almost all HCC samples (26/28; Figures 6B, 6C and Table S2). In contrast, unaffected liver tissue from HCC patients showed significantly less CHOP staining, and non-HCC liver samples showed less still (Figure 6B, 6C). The degree of CHOP staining did not correlate with any clinical measure of HCC, including patient gender, age, Hepatitis B, C or CMV status, cirrhosis, or tumor grade; rather, CHOP was a nearly uniform biomarker for HCC (Table S2). Dual staining for cell-type specific markers revealed that CHOP was expressed in hepatocytes, but not macrophages, bile ducts, or activated stellate cells within the liver (Figure 6D). These data indicate that CHOP expression broadly characterizes HCC tumors, and suggest that the impacts of CHOP on inflammatory gene expression and fibrosis are relevant to human HCC.

## Discussion

**Figure 7:**
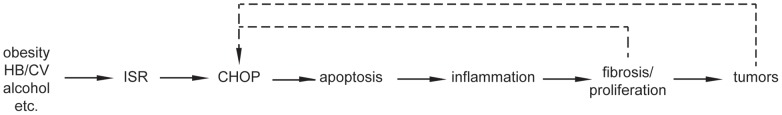
Model for the role of CHOP in HCC. The simplest interpretation of the data presented is a linear pathway whereby CHOP promotes apoptosis, which in turn activates inflammatory signaling, leading to fibrosis, compensatory proliferation, and ultimately tumorigenesis. Fibrosis and cellular transformation might in turn exacerbate cellular stress and CHOP expression, amplifying the tumor-promoting function of CHOP.

This is the first report to our knowledge that links CHOP to hepatocellular oncogenesis. Our data lead us to propose a working model for the role of CHOP in HCC (Figure 7): Hepatocyte injury, be it in the form of lipotoxicity associated with obesity, the toxicity of ethanol metabolism, viral hepatitis, or genotoxic challenge, leads to activation of the ISR and production of CHOP either directly or after the initial formation of preneoplastic lesions such as the foci of cellular alteration that constitute some of the nodules produced by DEN. CHOP is strongly functionally associated with cell death in multiple contexts [17]. Consistent with that function, there is less cell death in the nodules of DEN challenged *Chop*−/− mice than wild-type animals (Figure 3). CHOP might promote cell death by enhancing production of reactive oxygen species [8], although we as yet have found no evidence for altered ROS production in *Chop*−/− mice. Alternatively, CHOP-dependent cell death might also be provoked directly as a consequence of regulation of BCL-dependent pathways [45], [46], [47]. The mechanism by which CHOP promotes hepatocyte cell death will be the subject of future investigation.

We propose that initiation of cell death by CHOP provokes an inflammatory response, accounting for why *Chop*−/− mice show lower basal levels of inflammatory gene expression (Figure 5). Although classically thought to be “silent,” more recently apoptosis has been shown to stimulate inflammation through several mechanisms including failure to clear apoptotic bodies, activation of pro-inflammatory signals in Kupffer cells that engulf apoptotic bodies, and secretion of pro-inflammatory cytokines by the apoptotic cells themselves [48]. We propose that inflammation stimulates hepatic stellate cell activation and fibrotic deposition, which would account for attenuated fibrosis in DEN-challenged *Chop*−/− animals (Figure 4). Inflammation also promotes compensatory hepatocyte proliferation through the effects of mitogenic cytokines [38], the diffusible nature of which would explain why *Chop*−/− animals show fewer Ki-67- and PCNA-positive cells than wild-type animals, and why these cells, unlike caspase-3-positive cells, are more-or-less uniformly distributed throughout the liver (Figure 3). The enhanced fibrosis and proliferation are then more likely to stimulate tumor formation. It is also possible that inflammation and fibrosis might further stimulate CHOP production, amplifying the effects of CHOP on tumorigenesis.

This work establishes a functional role for CHOP in hepatic oncogenesis. While previous microarray-based approaches have not suggested *Chop* as a gene of interest ([49] and references therein), our starting focus on UPR- and ISR-regulated genes allowed us to identify and subsequently experimentally validate a potential role for CHOP and provide a plausible pathway by which this role might be realized. The next step will be to test the key causal relationships with higher temporal resolution. Our data in *Chop*−/− mice allow us to place CHOP upstream of both fibrosis and tumorigenesis, since these animals are partially protected from both. Thus, we favor a model whereby the progression from CHOP expression to liver tumors occurs in a stepwise fashion, because this view is consistent with the prevalent notions of the etiology of HCC. Yet the observation that tumors in wild-type versus *Chop*−/− animals can be distinguished on at least one basis—the expression of ribosomal genes—and that CHOP expression seems most predominantly associated with nodules and tumors rather than surrounding non-transformed tissue in both mice and humans suggests that CHOP exerts an ongoing influence on tumor biochemistry. The fact that numerous genes encoding ribosomal subunits are upregulated in tumors from *Chop*−/− mice would suggest that CHOP ultimately inhibits protein synthesis. However, during the canonical ER stress response, CHOP is known to promote protein synthesis through upregulation of the regulatory GADD34 subunit of the PP1 phosphatase that dephosphorylates eIF2α [26]. Thus, it will be necessary to determine whether protein synthesis is actually altered in tumors from *Chop*−/− mice, and whether such alteration contributes to the apparent retardation of tumor growth seen in these animals.

Our findings do not exclude the possibility that CHOP only assumes an aggravating role in fibrosis and tumorigenesis after the process has already begun. Or, alterntively, CHOP might promote the steps of inflammation, fibrosis, and tumorigenesis independently of each other. Further experiments will be needed to determine when CHOP is expressed during the tumorigenic process, and more particularly, whether its expression precedes the formation of neoplastic lesions and fibrotic deposition, and whether it is expressed in less- or more-advanced tumors. These—along with an inducible expression system for CHOP in the liver, which has not to our knowledge been reported—would enable us to determine whether CHOP facilitates fibrosis and initial tumor formation, or instead promotes further tumorigenesis once tumors have already formed; either possibility would be consistent with the data presented here.

It is not currently clear whether the CHOP-independent ISR pathways activated during transformation also promote tumor growth or act against it, nor is the identity of the ISR kinase activated during the process. The balance of data suggest that ISR activation is generally anti-tumorigenic [50], yet ISR activation can clearly promote tumor growth in at least certain instances [15], [51], [52]. Liver-specific manipulation of the eIF2α kinases (PERK, PKR, HRI, and GCN2) will allow their roles to be tested. Also of potential use in this respect is a recently described inhibitor of PERK [53], [54], which, by blunting CHOP upregulation, might either protect against or reverse HCC in mouse models. The approximately 1000-fold selectivity of this molecule for PERK over other eIF2α kinases [53] will also enable determination of whether PERK is required for CHOP upregulation in these models.

Another question to be addressed in future work is the cell autonomy of CHOP's pro-oncogenic effects. CHOP expression is associated with hepatocytes rather than macrophages, bile ducts, or stellate cells in human HCC (Figure 6), and appears to be so in mouse tumors as well (unpublished data). However, the immune and inflammatory genes that are differentially expressed in *Chop*−/− livers (Figure 5) are largely expressed in immune cells rather than hepatocytes. It thus seems most likely that CHOP acts cell-non-autonomously by promoting inflammation and compensatory proliferation. Or, CHOP dependence might arise entirely from CHOP function in a non-hepatocyte cell type (such as Kupffer cells), with tumor-associated CHOP expression being merely coincidental. Alternatively, the fact that TLS-CHOP and EWS-CHOP fusions show direct transformation potential leaves open the possibility that CHOP is directly transforming in hepatocytes as well. The transforming activity of fused CHOP in myxoid liposarcoma has been attributed to its ability to interfere with the activity of other transcription factors of the C/EBP family, thereby interfering with the adipogenic differentiation program, including expression of the inducing factor PPARγ2 [55]–[57]. Given the association of dysregulated lipid metabolism with HCC—particularly via the activity of PPAR family members [58]–CHOP could promote HCC through altered lipid metabolism in parallel to an inflammatory mechanism, or such alterations could be the upstream stimulus that induces inflammation. Consistent with this idea, we recently demonstrated that CHOP alters expression of lipid metabolic genes including *Ppara* through interactions with C/EBP family members [59]. Or, CHOP might act cell-autonomously to de-differentiate cells and thereby loosen the constraints on their proliferative capacity. Ultimately, tissue-specific manipulation of CHOP expression will be required to discriminate among these hypotheses. CHOP expression is correlated with poor prognosis in both colorectal cancer and mesothelioma [60], [61] and elevated CHOP expression was seen in the central hypoxic regions of human cervical tumors [51]. Thus, understanding the mechanisms, both direct and indirect, by which CHOP promotes oncogenesis will likely have implications for other cancers beyond HCC.

Our results have revealed in CHOP an unexpected molecular link between hepatocarcinogenesis and the stimuli that predispose to it. Understanding how CHOP is induced by these stimuli and how it ultimately promotes tumorigenesis will suggest additional points for potential therapeutic intervention, which are sorely needed for HCC.

Note added in proof: It has come to our attention that, consistent with the results shown here, another group has found that *Chop*−/− mice are resistant to chemical hepatocarcinogenesis [62].

## Materials and Methods

### Ethics Statement

All protocols for animal use were reviewed and approved by the University Committee on Use and Care of Animals at the University of Iowa, including provisions for minimizing animal distress and maximizing animal well-being that conform to NIH guidelines. Animals were fed standard rodent chow and housed in a controlled environment with 12 hr light and dark cycles. Human tissue and associated clinical information were obtained from de-identified samples, kept by the University of Iowa Department of Pathology. Analysis was IRB-exempt.

### Animal Experiments

*Chop*−/− mice have been backcrossed in-house for >10 generations into the C57Bl/6J line. Generation of HCCs from T2/Onc3-expressing mice has been described [24].

For DEN treatment, 15 d.o. C57Bl/6J and *Chop*−/− male mice were injected once intraperitoneally (i.p.) with 25 mg/kg DEN (Sigma) or PBS. The mice were sacrificed at 9 months of age. Externally visible nodules and large tumors were counted blinded to genotype. Portions of the liver and of large tumors were either fixed in 10% formalin or snap frozen. Nodule sectional surface area was quantitated in Image J.

For CCl_4_ treatment, 8 week-old male wild-type or T2/Onc3-expressing mice were given i.p. injections of 10% CCl_4_ in mineral oil. Mice were injected with 2.5 µl/g b.w. twice a week for a total of twelve weeks.

### Histology

IHC was performed with the GADD153/CHOP (Santa Cruz, sc-7351); BiP (BD Biosciences, 610978); Ki-67 (Epitomics, 4203-1) or PCNA (Dako, M0879) antibodies, using the M.O.M. kit for detection (Vector Labs). Double staining was performed using antibodies against cytokeratin 7 (Epitomics, 2303-1), cytokeratin 18 (Epitomics, 3258-1), CD68 (Epitomics, 2135-1), or SMA (Abcam, ab5694) in conjunction with the GADD153 antibody using the Tandem Dual Staining kit (Epitomics). Cleaved Caspase-3 immunostaining was performed using the SignalStain Cleaved Caspase-3 (Asp175) IHC Detection Kit (Cell Signaling). Sections were visualized using a Nikon light microscope with a 10× or 20× objective.

All histological scoring was performed blinded to genotype. The level of fibrosis was scored using the METAVIR scoring system. For each sample, six pictures at different locations within the sample were taken and individually scored. The six scores were averaged to generate final scores for the individual samples, and were analyzed for significance by a Wilcoxon rank sum test. Ki-67 staining was scored as follows: A score of 0 indicated between 0 to 10 Ki-67-positive nuclei stained per field of view; 1, 11 to 40; 2, 41 to 100; and 3, over 100. PCNA scoring was similar: 0: 0–5 PCNA-positive nuclei per field; 1: 6–20; 2: 21–50; and 3: 51+. Both Ki-67 and PCNA scoring were grouped and statistically analyzed as for fibrotic scoring.

### Molecular Analysis

Transcriptome sequencing was performed as described, and the raw data provided as supplemental material therein [25]. Primer sequences for qRT-PCR analysis, performed as described [63], are listed in Table S3. We normalized qRT-PCR expression against two housekeeping genes rather than one to help ensure that changes in expression depended upon the mRNAs being analyzed rather than the housekeeping genes; for similar reasons we used *Btf3* and *Ppia* as normalizers because we have found their expression to be more uniform and more linear across a wider dynamic range than more commonly used normalizers such as *18s* rRNA, *actin*, or *Gapdh* (unpublished observations). Microarrays were performed by the University of Iowa DNA Core, starting from 100 ng of total RNA from livers of 9-month-old PBS-treated wild-type (n = 3) or *Chop*−/− (n = 3) animals, large tumors from DEN-treated wild-type (n = 3) or *Chop*−/− (n = 2) animals, or livers of 9-month-old DEN-treated wild-type (n = 6) or *Chop*−/− (n = 7) animals. Illumina MouseRef-8 v2.0 BeadChips were used according to the manufacturer's protocol. Hybridized arrays were stained with streptavidin-Cy3 (GE Healthcare, Piscataway, NJ). BeadChips were scanned with the Illumina iScan system, and data collected using GenomeStudio software v2011.1. Normalized expression data are given in Table S4. The NCBI GEO accession number for microarray data is GSE51188. Pathway analysis was performed with DAVID.

## Supporting Information

Figure S1 Further mRNA expression characterization in normal liver tissue and SB-derived tumors. (A) The expression of 9 genes previously described as ATF4-dependent (Harding et al. (2003) *Mol Cell* 11, 619) was assessed from the transcriptome analysis described in Figure 1A. Expression is given in log_2_-transformed terms. (B) *Chop* expression by qRT-PCR of each individual normal or tumor sample from Figure 1B is shown.
(PDF)

Figure S2 IHC antibody specifically detects CHOP in mouse liver. Livers from wild-type or *Chop*−/− mice were resected 8 hours after injection with PBS or 1 mg/kg of the ER stress-inducing agent tunicamycin (TM). They were then fixed in formalin and probed for CHOP expression by IHC.
(PDF)

Figure S3 Suppression of immune and inflammatory genes in DEN-treated *Chop*−/− livers. mRNA expression from livers of 9 month-old DEN-treated wild-type (n = 6) or *Chop*−/− (n = 7) animals were analyzed by microarray as in Figure 5. Because these samples included both nodular and uninvolved tissue, gene expression levels were considerably more heterogeneous. Nevertheless, of the 19 downregulated genes shown in Figure 5C, 8 were also significantly downregulated in *Chop*−/− DEN-treated livers, one (*Clec7a*) was near the threshold of significance (p∼0.06) and none was significantly upregulated.
(PDF)

Figure S4 IHC antibody specifically detects CHOP in human cells. Human alveolar epithelial A549 cells were treated with the ER stress-inducing agent thapsigargin (TG; 500 nM) for 16 hours, and CHOP was detected by immunofluorescence. Brightfield images show the same fields of view. This result establishes the specificity of the CHOP antibody in human cells.
(PDF)

Table S1 Genes differentially regulated in untreated wild-type versus *Chop*−/− livers.
(PDF)

Table S2 CHOP positivity with relationship to patient status.
(PDF)

Table S3 Primer sequences used.
(PDF)

Table S4 Normalized log_2_-transformed Microarray data using Illumina MouseRef-8 v.2.0 BeadChips.
(XLSX)

## References

[1] Center MM, Jemal A (2011) International trends in liver cancer incidence rates. Cancer Epidemiol Biomarkers Prev 20: 2362–2368. 21921256 10.1158/1055-9965.EPI-11-0643

[2] Altekruse SF, McGlynn KA, Reichman ME (2009) Hepatocellular carcinoma incidence, mortality, and survival trends in the United States from 1975 to 2005. J Clin Oncol 27: 1485–1491. 19224838 10.1200/JCO.2008.20.7753 PMC2668555

[3] El-Serag HB, Rudolph KL (2007) Hepatocellular carcinoma: epidemiology and molecular carcinogenesis. Gastroenterology 132: 2557–2576. 17570226 10.1053/j.gastro.2007.04.061

[4] Shen C, Zhao CY, Zhang R, Qiao L (2012) Obesity-related hepatocellular carcinoma: roles of risk factors altered in obesity. Front Biosci 17: 2356–2370. 10.2741/4057 22652784

[5] Starley BQ, Calcagno CJ, Harrison SA (2010) Nonalcoholic fatty liver disease and hepatocellular carcinoma: a weighty connection. Hepatology 51: 1820–1832. 20432259 10.1002/hep.23594

[6] Rutkowski DT, Hegde RS (2010) Regulation of basal cellular physiology by the homeostatic unfolded protein response. J Cell Biol 189: 783–794. 20513765 10.1083/jcb.201003138 PMC2878945

[7] Harding HP, Novoa I, Zhang Y, Zeng H, Wek R, et al (2000) Regulated translation initiation controls stress-induced gene expression in mammalian cells. Mol Cell 6: 1099–1108. 11106749 10.1016/s1097-2765(00)00108-8

[8] Walter P, Ron D (2011) The unfolded protein response: from stress pathway to homeostatic regulation. Science 334: 1081–1086. 22116877 10.1126/science.1209038

[9] Harding HP, Zhang Y, Zeng H, Novoa I, Lu PD, et al (2003) An integrated stress response regulates amino acid metabolism and resistance to oxidative stress. Mol Cell 11: 619–633. 12667446 10.1016/s1097-2765(03)00105-9

[10] Dong D, Ni M, Li J, Xiong S, Ye W, et al (2008) Critical role of the stress chaperone GRP78/BiP in tumor proliferation, survival, and tumor angiogenesis in transgene-induced mammary tumor development. Cancer Res 68: 498–505. 18199545 10.1158/0008-5472.CAN-07-2950

[11] Ashktorab H, Green W, Finzi G, Sessa F, Nouraie M, et al (2012) SEL1L, an UPR response protein, a potential marker of colonic cell transformation. Dig Dis Sci 57: 905–912. 22350780 10.1007/s10620-011-2026-y PMC3345950

[12] So AY, de la Fuente E, Walter P, Shuman M, Bernales S (2009) The unfolded protein response during prostate cancer development. Cancer Metastasis Rev 28: 219–223. 19172382 10.1007/s10555-008-9180-5

[13] Pyrko P, Schonthal AH, Hofman FM, Chen TC, Lee AS (2007) The unfolded protein response regulator GRP78/BiP as a novel target for increasing chemosensitivity in malignant gliomas. Cancer Res 67: 9809–9816. 17942911 10.1158/0008-5472.CAN-07-0625

[14] Bartkowiak K, Effenberger KE, Harder S, Andreas A, Buck F, et al (2010) Discovery of a novel unfolded protein response phenotype of cancer stem/progenitor cells from the bone marrow of breast cancer patients. J Proteome Res 9: 3158–3168. 20423148 10.1021/pr100039d

[15] Bobrovnikova-Marjon E, Grigoriadou C, Pytel D, Zhang F, Ye J, et al (2010) PERK promotes cancer cell proliferation and tumor growth by limiting oxidative DNA damage. Oncogene 29: 3881–3895. 20453876 10.1038/onc.2010.153 PMC2900533

[16] Shuda M, Kondoh N, Imazeki N, Tanaka K, Okada T, et al (2003) Activation of the ATF6, XBP1 and grp78 genes in human hepatocellular carcinoma: a possible involvement of the ER stress pathway in hepatocarcinogenesis. J Hepatol 38: 605–614. 12713871 10.1016/s0168-8278(03)00029-1

[17] Oyadomari S, Mori M (2004) Roles of CHOP/GADD153 in endoplasmic reticulum stress. Cell Death Differ 11: 381–389. 14685163 10.1038/sj.cdd.4401373

[18] Schonthal AH (2009) Endoplasmic reticulum stress and autophagy as targets for cancer therapy. Cancer Lett 275: 163–169. 18692955 10.1016/j.canlet.2008.07.005

[19] Moon DO, Park SY, Choi YH, Ahn JS, Kim GY (2011) Guggulsterone sensitizes hepatoma cells to TRAIL-induced apoptosis through the induction of CHOP-dependent DR5: involvement of ROS-dependent ER-stress. Biochem Pharmacol 82: 1641–1650. 21903093 10.1016/j.bcp.2011.08.019

[20] Crozat A, Aman P, Mandahl N, Ron D (1993) Fusion of CHOP to a novel RNA-binding protein in human myxoid liposarcoma. Nature 363: 640–644. 8510758 10.1038/363640a0

[21] Rabbitts TH, Forster A, Larson R, Nathan P (1993) Fusion of the dominant negative transcription regulator CHOP with a novel gene FUS by translocation t(12;16) in malignant liposarcoma. Nat Genet 4: 175–180. 7503811 10.1038/ng0693-175

[22] Panagopoulos I, Hoglund M, Mertens F, Mandahl N, Mitelman F, et al (1996) Fusion of the EWS and CHOP genes in myxoid liposarcoma. Oncogene 12: 489–494. 8637704

[23] Dupuy AJ (2010) Transposon-based screens for cancer gene discovery in mouse models. Semin Cancer Biol 20: 261–268. 20478384 10.1016/j.semcancer.2010.05.003 PMC2940989

[24] Dupuy AJ, Rogers LM, Kim J, Nannapaneni K, Starr TK, et al (2009) A modified sleeping beauty transposon system that can be used to model a wide variety of human cancers in mice. Cancer Res 69: 8150–8156. 19808965 10.1158/0008-5472.CAN-09-1135 PMC3700628

[25] Riordan JD, Keng VW, Tschida BR, Scheetz TE, Bell JB, et al (2013) Identification of rtl1, a retrotransposon-derived imprinted gene, as a novel driver of hepatocarcinogenesis. PLoS Genet 9: e1003441. 23593033 10.1371/journal.pgen.1003441 PMC3616914

[26] Marciniak SJ, Yun CY, Oyadomari S, Novoa I, Zhang Y, et al (2004) CHOP induces death by promoting protein synthesis and oxidation in the stressed endoplasmic reticulum. Genes Dev 18: 3066–3077. 15601821 10.1101/gad.1250704 PMC535917

[27] Ohoka N, Yoshii S, Hattori T, Onozaki K, Hayashi H (2005) TRB3, a novel ER stress-inducible gene, is induced via ATF4-CHOP pathway and is involved in cell death. Embo J 24: 1243–1255. 15775988 10.1038/sj.emboj.7600596 PMC556400

[28] Lee AH, Iwakoshi NN, Glimcher LH (2003) XBP-1 regulates a subset of endoplasmic reticulum resident chaperone genes in the unfolded protein response. Mol Cell Biol 23: 7448–7459. 14559994 10.1128/MCB.23.21.7448-7459.2003 PMC207643

[29] Wu J, Rutkowski DT, Dubois M, Swathirajan J, Saunders T, et al (2007) ATF6alpha optimizes long-term endoplasmic reticulum function to protect cells from chronic stress. Dev Cell 13: 351–364. 17765679 10.1016/j.devcel.2007.07.005

[30] Zinszner H, Kuroda M, Wang X, Batchvarova N, Lightfoot RT, et al (1998) CHOP is implicated in programmed cell death in response to impaired function of the endoplasmic reticulum. Genes Dev 12: 982–995. 9531536 10.1101/gad.12.7.982 PMC316680

[31] Starkel P, Leclercq IA (2011) Animal models for the study of hepatic fibrosis. Best Pract Res Clin Gastroenterol 25: 319–333. 21497748 10.1016/j.bpg.2011.02.004

[32] Vesselinovitch SD, Mihailovich N (1983) Kinetics of diethylnitrosamine hepatocarcinogenesis in the infant mouse. Cancer Res 43: 4253–4259. 6871863

[33] Maeda S, Kamata H, Luo JL, Leffert H, Karin M (2005) IKKbeta couples hepatocyte death to cytokine-driven compensatory proliferation that promotes chemical hepatocarcinogenesis. Cell 121: 977–990. 15989949 10.1016/j.cell.2005.04.014

[34] Baffy G, Brunt EM, Caldwell SH (2012) Hepatocellular carcinoma in non-alcoholic fatty liver disease: an emerging menace. J Hepatol 56: 1384–1391. 22326465 10.1016/j.jhep.2011.10.027

[35] Tamaki N, Hatano E, Taura K, Tada M, Kodama Y, et al (2008) CHOP deficiency attenuates cholestasis-induced liver fibrosis by reduction of hepatocyte injury. Am J Physiol Gastrointest Liver Physiol 294: G498–505. 18174271 10.1152/ajpgi.00482.2007

[36] Malhi H, Kropp EM, Clavo VF, Kobrossi CR, Han J, et al (2013) C/EBP Homologous Protein-induced Macrophage Apoptosis Protects Mice from Steatohepatitis. J Biol Chem 288: 18624–18642. 23720735 10.1074/jbc.M112.442954 PMC3696637

[37] Sakurai T, Maeda S, Chang L, Karin M (2006) Loss of hepatic NF-kappa B activity enhances chemical hepatocarcinogenesis through sustained c-Jun N-terminal kinase 1 activation. Proc Natl Acad Sci U S A 103: 10544–10551. 16807293 10.1073/pnas.0603499103 PMC1502270

[38] Sun B, Karin M (2012) Obesity, inflammation, and liver cancer. J Hepatol 56: 704–713. 22120206 10.1016/j.jhep.2011.09.020 PMC3889660

[39] Zheng JF, Liang LJ (2008) Intra-portal transplantation of bone marrow stromal cells ameliorates liver fibrosis in mice. Hepatobiliary Pancreat Dis Int 7: 264–270. 18522880

[40] Qi Z, Atsuchi N, Ooshima A, Takeshita A, Ueno H (1999) Blockade of type beta transforming growth factor signaling prevents liver fibrosis and dysfunction in the rat. Proc Natl Acad Sci U S A 96: 2345–2349. 10051644 10.1073/pnas.96.5.2345 PMC26786

[41] Schiffer E, Housset C, Cacheux W, Wendum D, Desbois-Mouthon C, et al (2005) Gefitinib, an EGFR inhibitor, prevents hepatocellular carcinoma development in the rat liver with cirrhosis. Hepatology 41: 307–314. 15660382 10.1002/hep.20538

[42] Jenkins SA, Grandison A, Baxter JN, Day DW, Taylor I, et al (1985) A dimethylnitrosamine-induced model of cirrhosis and portal hypertension in the rat. J Hepatol 1: 489–499. 4056351 10.1016/s0168-8278(85)80747-9

[43] Bataller R, Brenner DA (2005) Liver fibrosis. J Clin Invest 115: 209–218. 15690074 10.1172/JCI24282 PMC546435

[44] Han J, Back SH, Hur J, Lin YH, Gildersleeve R, et al (2013) ER-stress-induced transcriptional regulation increases protein synthesis leading to cell death. Nat Cell Biol 15: 481–490. 23624402 10.1038/ncb2738 PMC3692270

[45] McCullough KD, Martindale JL, Klotz LO, Aw TY, Holbrook NJ (2001) Gadd153 sensitizes cells to endoplasmic reticulum stress by down-regulating Bcl2 and perturbing the cellular redox state. Mol Cell Biol 21: 1249–1259. 11158311 10.1128/MCB.21.4.1249-1259.2001 PMC99578

[46] Puthalakath H, O'Reilly LA, Gunn P, Lee L, Kelly PN, et al (2007) ER stress triggers apoptosis by activating BH3-only protein Bim. Cell 129: 1337–1349. 17604722 10.1016/j.cell.2007.04.027

[47] Zagorodna O, Martin SM, Rutkowski DT, Kuwana T, Spitz DR, et al (2011) 2-Deoxyglucose-induced toxicity is regulated by Bcl-2 family members and is enhanced by antagonizing Bcl-2 in lymphoma cell lines. Oncogene 31: 2738–2749. 21986940 10.1038/onc.2011.454 PMC3257357

[48] Canbay A, Friedman S, Gores GJ (2004) Apoptosis: the nexus of liver injury and fibrosis. Hepatology 39: 273–278. 14767974 10.1002/hep.20051

[49] Maass T, Sfakianakis I, Staib F, Krupp M, Galle PR, et al (2010) Microarray-based gene expression analysis of hepatocellular carcinoma. Curr Genomics 11: 261–268. 21119890 10.2174/138920210791233063 PMC2930665

[50] Dalton LE, Healey E, Irving J, Marciniak SJ (2012) Phosphoproteins in stress-induced disease. Prog Mol Biol Transl Sci 106: 189–221. 22340719 10.1016/B978-0-12-396456-4.00003-1

[51] Bi M, Naczki C, Koritzinsky M, Fels D, Blais J, et al (2005) ER stress-regulated translation increases tolerance to extreme hypoxia and promotes tumor growth. Embo J 24: 3470–3481. 16148948 10.1038/sj.emboj.7600777 PMC1276162

[52] Blais JD, Addison CL, Edge R, Falls T, Zhao H, et al (2006) Perk-dependent translational regulation promotes tumor cell adaptation and angiogenesis in response to hypoxic stress. Mol Cell Biol 26: 9517–9532. 17030613 10.1128/MCB.01145-06 PMC1698539

[53] Axten JM, Medina JR, Feng Y, Shu A, Romeril SP, et al (2012) Discovery of 7-methyl-5-(1-{[3-(trifluoromethyl)phenyl]acetyl}-2,3-dihydro-1H-indol-5-yl)-7H-p yrrolo[2,3-d]pyrimidin-4-amine (GSK2606414), a potent and selective first-in-class inhibitor of protein kinase R (PKR)-like endoplasmic reticulum kinase (PERK). J Med Chem 55: 7193–7207. 22827572 10.1021/jm300713s

[54] Harding HP, Zyryanova AF, Ron D (2012) Uncoupling proteostasis and development in vitro with a small molecule inhibitor of the pancreatic endoplasmic reticulum kinase, PERK. J Biol Chem 287: 44338–44344. 23148209 10.1074/jbc.M112.428987 PMC3531748

[55] Adelmant G, Gilbert JD, Freytag SO (1998) Human translocation liposarcoma-CCAAT/enhancer binding protein (C/EBP) homologous protein (TLS-CHOP) oncoprotein prevents adipocyte differentiation by directly interfering with C/EBPbeta function. J Biol Chem 273: 15574–15581. 9624148 10.1074/jbc.273.25.15574

[56] Clarke SL, Robinson CE, Gimble JM (1997) CAAT/enhancer binding proteins directly modulate transcription from the peroxisome proliferator-activated receptor gamma 2 promoter. Biochem Biophys Res Commun 240: 99–103. 9367890 10.1006/bbrc.1997.7627

[57] Batchvarova N, Wang XZ, Ron D (1995) Inhibition of adipogenesis by the stress-induced protein CHOP (Gadd153). Embo J 14: 4654–4661. 7588595 10.1002/j.1460-2075.1995.tb00147.x PMC394562

[58] Ockner RK, Kaikaus RM, Bass NM (1993) Fatty-acid metabolism and the pathogenesis of hepatocellular carcinoma: review and hypothesis. Hepatology 18: 669–676. 8395460

[59] Chikka MR, McCabe DD, Tyra HM, Rutkowski DT (2013) C/EBP homologous protein (CHOP) contributes to suppression of metabolic genes during endoplasmic reticulum stress in the liver. J Biol Chem 288: 4405–4415. 23281479 10.1074/jbc.M112.432344 PMC3567690

[60] Rask K, Thorn M, Ponten F, Kraaz W, Sundfeldt K, et al (2000) Increased expression of the transcription factors CCAAT-enhancer binding protein-beta (C/EBBeta) and C/EBzeta (CHOP) correlate with invasiveness of human colorectal cancer. Int J Cancer 86: 337–343. 10760820 10.1002/(sici)1097-0215(20000501)86:3<337::aid-ijc6>3.0.co;2-3

[61] Dalton LE, Clarke HJ, Knight J, Lawson MH, Wason J, et al (2013) The endoplasmic reticulum stress marker CHOP predicts survival in malignant mesothelioma. Br J Cancer 108: 1340–1347. 23412101 10.1038/bjc.2013.66 PMC3619254

[62] Scaiewicz V, Nahmias A, Chung RT, Mueller T, Tirosh B, et al (2013) CCAAT/Enhancer-Binding Protein omologous (CHOP) Protein Promotes Carcinogenesis in the DEN-Induced Hepatocellular Carcinoma Model. PLoS ONE 8(12): e81065 doi:10.1371/journal.pone.0081065 24339898 10.1371/journal.pone.0081065 PMC3855209

[63] Rutkowski DT, Arnold SM, Miller CN, Wu J, Li J, et al (2006) Adaptation to ER stress is mediated by differential stabilities of pro-survival and pro-apoptotic mRNAs and proteins. PLoS Biol 4: e374. 17090218 10.1371/journal.pbio.0040374 PMC1634883

