# Supplementary figures and images for: The Stress-Regulated Transcription Factor CHOP Promotes Hepatic Inflammatory Gene Expression, Fibrosis, and Oncogenesis
Source: PLoS Genet. 2013 Dec 19;9(12):e1003937. doi: 10.1371/journal.pgen.1003937 (PMC3868529; doi:10.1371/journal.pgen.1003937)

A

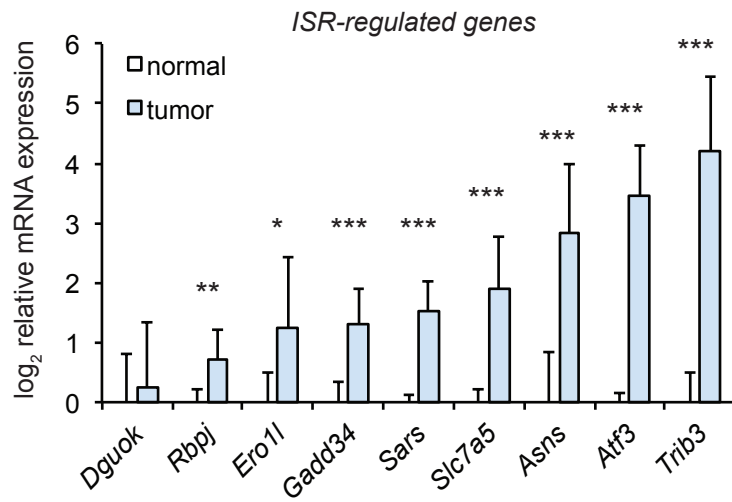

B

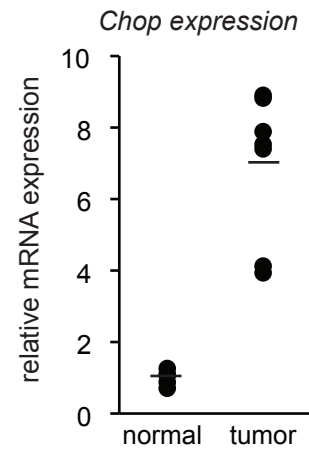

Supplement: Figure S1 — Further mRNA expression characterization in normal liver tissue and SB-derived tumors. (A) The expression of 9 genes previously described as ATF4-dependent (Harding et al. (2003) Mol Cell 11, 619) was assessed from the transcriptome analysis described in Figure 1A. Expression is given in log2-transformed terms. (B) Chop expression by qRT-PCR of each individual normal or tumor sample from Figure 1B is shown. (PDF) [file pgen.1003937.s001.pdf]

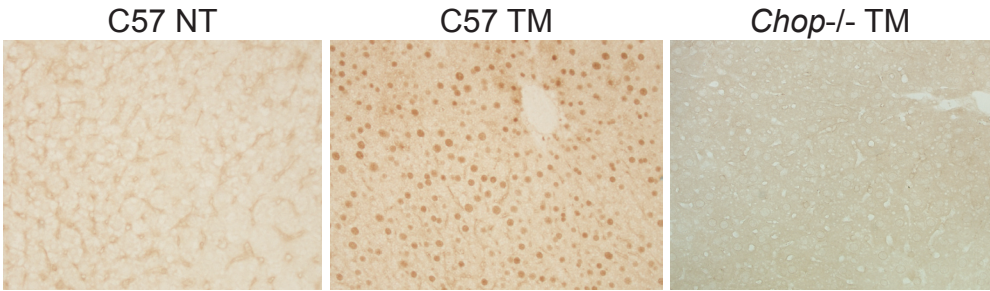

Supplement: Figure S2 — IHC antibody specifically detects CHOP in mouse liver. Livers from wild-type or Chop−/− mice were resected 8 hours after injection with PBS or 1 mg/kg of the ER stress-inducing agent tunicamycin (TM). They were then fixed in formalin and probed for CHOP expression by IHC. (PDF) [file pgen.1003937.s002.pdf]

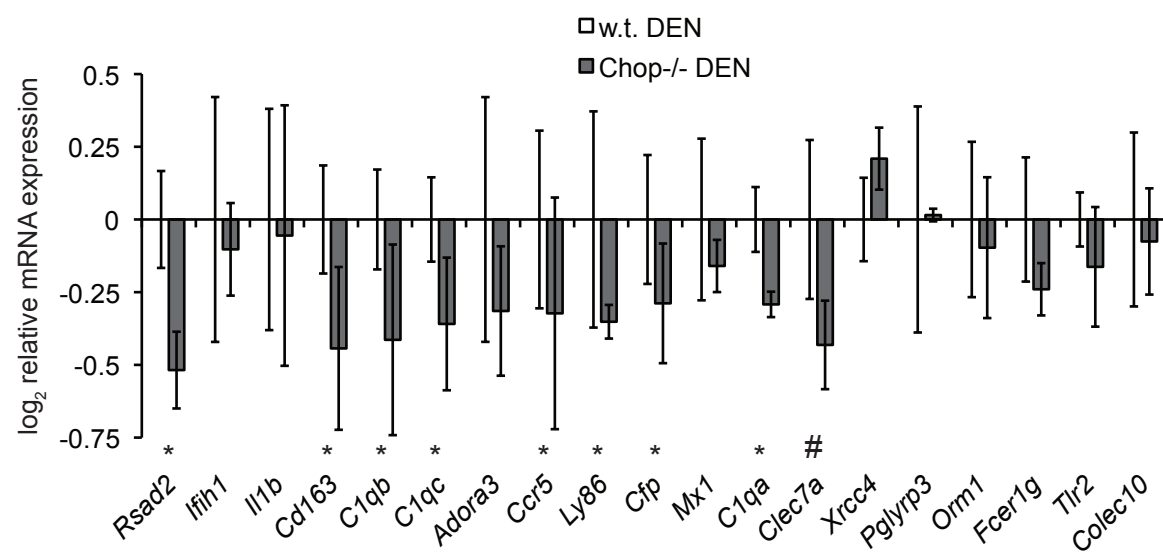

Supplement: Figure S3 — Suppression of immune and inflammatory genes in DEN-treated Chop−/− livers. mRNA expression from livers of 9 month-old DEN-treated wild-type (n = 6) or Chop−/− (n = 7) animals were analyzed by microarray as in Figure 5. Because these samples included both nodular and uninvolved tissue, gene expression levels were considerably more heterogeneous. Nevertheless, of the 19 downregulated genes shown in Figure 5C, 8 were also significantly downregulated in Chop−/− DEN-treated livers, one (Clec7a) was near the threshold of significance (p∼0.06) and none was significantly upregulated. (PDF) [file pgen.1003937.s003.pdf]

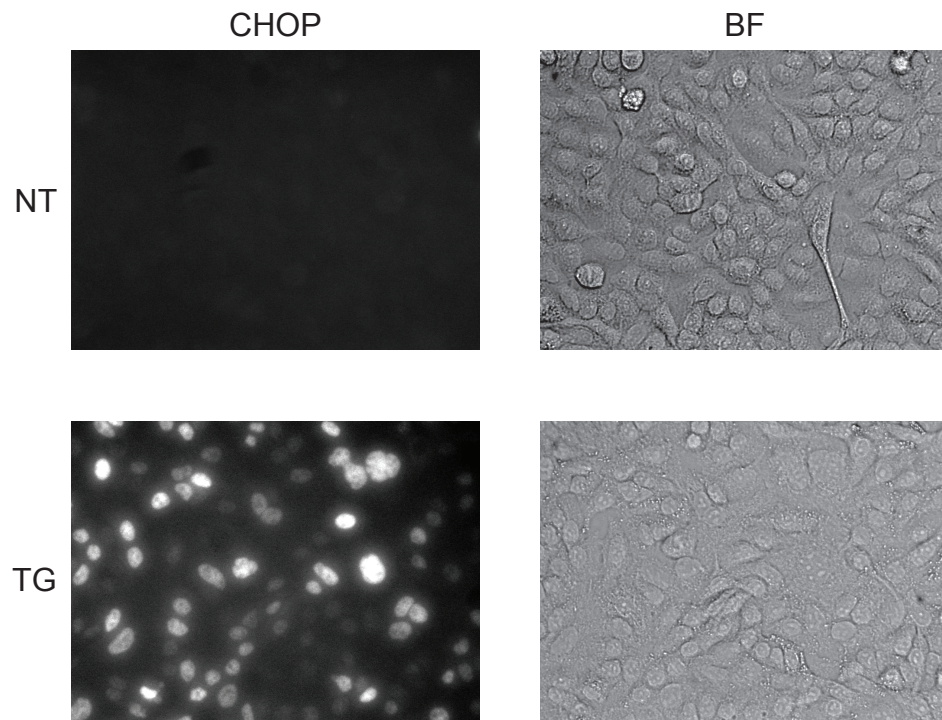

Supplement: Figure S4 — IHC antibody specifically detects CHOP in human cells. Human alveolar epithelial A549 cells were treated with the ER stress-inducing agent thapsigargin (TG; 500 nM) for 16 hours, and CHOP was detected by immunofluorescence. Brightfield images show the same fields of view. This result establishes the specificity of the CHOP antibody in human cells. (PDF) [file pgen.1003937.s004.pdf]
