# Supplementary material for: The Stress-Regulated Transcription Factor CHOP Promotes Hepatic Inflammatory Gene Expression, Fibrosis, and Oncogenesis
Source: PLoS Genet. 2013 Dec 19;9(12):e1003937. doi: 10.1371/journal.pgen.1003937 (PMC3868529; doi:10.1371/journal.pgen.1003937)
Supplement: Table S1 — Genes differentially regulated in untreated wild-type versus Chop−/− livers. (PDF) [file pgen.1003937.s005.pdf]

Table S1: Genes differentially regulated in untreated wild-type versus Chop-/- livers

**All regulated genes**			
**Term**	**Genes**	**Fold Enrichment**	**Bonferroni**
GO:0006952~defense response	*IL18R1, IFIH1, LYZ2, ADORA3, H2-M3, DEFB39, LY86, LOC100044702, TLR2, COLEC10, RSAD2, IFI47, C1QC, CD163, CFP, C1QA, ORM1, C1QB, CCR5, PGLYRP3, FCER1G, IL1B, CLEC7A, MX1*	3.3	0.001482
GO:0006955~immune response	*XRCC4, IL18R1, IFIH1, ADORA3, H2-M3, LY86, LOC100044702, TLR2, RSAD2, C1QC, CFP, C1QA, C1QB, CCR5, OASL2, PGLYRP3, FCER1G, IL1B, OAS1A, CLEC7A, PTMS, MX1*	2.9	0.037384

**Downregulated genes only**			
**Term**	**Genes**	**Fold Enrichment**	**Bonferroni**
GO:0006952~defense response	*IFIH1, LYZ2, ADORA3, H2-M3, LY86, COLEC10, TLR2, RSAD2, IFI47, C1QC, CD163, CFP, C1QA, ORM1, C1QB, CCR5, PGLYRP3, FCER1G, IL1B, CLEC7A, MX1*	5.3	0.000002
GO:0006955~immune response	*XRCC4, IFIH1, ADORA3, H2-M3, LY86, TLR2, RSAD2, C1QC, CFP, C1QA, C1QB, CCR5, OASL2, PGLYRP3, FCER1G, IL1B, OAS1A, CLEC7A, MX1*	4.6	0.000144
GO:0006954~inflammatory response	*C1QA, CFP, ORM1, C1QB, ADORA3, CCR5, LY86, TLR2, IL1B, CLEC7A, C1QC, CD163*	6.0	0.004636
GO:0002526~acute inflammatory response	*C1QA, CFP, ORM1, C1QB, ADORA3, IL1B, C1QC, CD163*	11.2	0.007059
GO:0009611~response to wounding	*ADORA3, LY86, TLR2, EVL, C1QC, CD163, C1QA, CFP, ORM1, C1QB, THBD, CCR5, IL1B, CLEC7A*	4.6	0.011191
GO:0048584~positive regulation of response to stimulus	*C1QA, CFP, C1QB, ADORA3, H2-M3, TLR2, FCER1G, IL1B, CLEC7A, C1QC*	6.1	0.038049
GO:0045087~innate immune response	*C1QA, CFP, C1QB, IFIH1, TLR2, CLEC7A, MX1, C1QC*	8.5	0.043095
