# Supplementary material for: The Stress-Regulated Transcription Factor CHOP Promotes Hepatic Inflammatory Gene Expression, Fibrosis, and Oncogenesis
Source: PLoS Genet. 2013 Dec 19;9(12):e1003937. doi: 10.1371/journal.pgen.1003937 (PMC3868529; doi:10.1371/journal.pgen.1003937)
Supplement: Table S2 — CHOP positivity with relationship to patient status. (PDF) [file pgen.1003937.s006.pdf]

Table S2: CHOP positivity with relationship to patient status

Sex	Age	CHOP	Hepatitis C	Cirrhosis	CMV	Grade	Etc.
M	19	+++	unk	Non-cirrhotic	unk	T2N1M0	
M	54	+++	Yes	Yes	No	T1N0M0	
M	75	+++	unk	Non-cirrhotic	unk	T3N0M0	
M	55	+++	Yes	Yes	unk	T1M0N0	
M	66	+++	No	Yes	pos	T1N0M0	
M	63	+++	No	Yes	No	T2N0M0	
M	63	+++	No	Yes	No	T2N0M0	
M	56	+++	No	Yes	No	T1N0M0	
F	52	+++	Yes	Yes	Yes	T1N0M0	Hep B+
F	57	++	unk	Non-cirrhotic	unk	T2N0M0	
F	62	++	No	Non-cirrhotic	No	T2N0M0	
M	67	++	Yes	Yes	unk	T1N0M0	
M	65	++	No	Yes	No	T1N0M0	
F	51	++	Yes	Yes	No	T2N0M0	
F	22	++	unk	Non-cirrhotic	unk	T3N1M1	
F	84	++	unk	Non-cirrhotic	unk	T3N0M0	
M	62	+	No	Non-cirrhotic	unk	T3N0M0	
M	61	+	Yes	Yes	unk	T3N0M0	
M	69	+	No	Non-cirrhotic	unk	T4N0M1	
F	80	+	No	Yes	No	T3N0M0	
M	52	+/-	Yes	Yes	pos	T2N0M0	
M	75	+/-	No	Non-cirrhotic	Yes	T1NXMX	
M	68	+/-	unk	Yes	unk	T2N0M0	Hep B+
M	54	+/-	No	Yes	No	T2N0M0	
M	74	+/-	unk	Non-cirrhotic	unk	T2N0M0	
M	61	+/-	Yes	Yes	No	T2N0M0	
M	67	-	Yes	Non-cirrhotic	unk	T1N1M1	Hep B+
F	61	-	unk	Non-cirrhotic	unk	T2M0N0	

+++ 15-20% positive nuclei
++ 10-14% positive nuclei
+ 5-9% positive nuclei
+/- 1-4% positive nuclei
- 0 positive nuclei
T = Pathological Primary Tumor
N = Regional Lymph Nodes
M = Distant Metastasis
