# Supplementary material for: The Stress-Regulated Transcription Factor CHOP Promotes Hepatic Inflammatory Gene Expression, Fibrosis, and Oncogenesis
Source: PLoS Genet. 2013 Dec 19;9(12):e1003937. doi: 10.1371/journal.pgen.1003937 (PMC3868529; doi:10.1371/journal.pgen.1003937)
Supplement: Table S3 — Primer sequences used. (PDF) [file pgen.1003937.s007.pdf]

Table S3: Primer sequences used

p58IPK	5'-TCCTGGTGGACCTGCAGTACG-3'
	5'-CTGCGAGTAATTTCTTCCCC-3'
Erdj3	5'-CCTTTATGGAGGCACTTCG-3'
	5'-GAACCTGAGCACCTTCTG-3'
Bip	5'-CATGGTTCTCACTAAAATGAAGG-3'
	5'-GCTGGTACAGTAACAACTG-3'
Chop	5'-CTGCCTTTCACCTTGGAGAC-3'
	5'-CGTTTCCTGGGGATGAGATA-3'
Edem1	5'-AAGTCTCAGGAGCTCAGAGTCATTAA-3'
	5'-CGATCTGGCGCATGTAGATG-3'
Herpud1	5'-AGCAGCCGGACAACTCTAAT-3'
	5'-CTTGGAAAGTCTGCTGGACA-3'
Xbp1 (tot)	5'-GAGCAGCAAGTGGTGGATTT-3'
	5'-CCGTGAGTTTTCTCCCGATAA-3'
Xbp1 (spl)	5'-GAGTCCGCAGCAGGTG-3'
	5'-GTGTCAGAGTCCATGGGA-3'
Wars	5'-CCTTGGACTACACAGCCAGGA-3'
	5'-CTAGGACCGAGGCCTGCAG-3'
Btf3	5'-AATGGTGAAGGTGTTTGCTG-3'
(housekeeping)	5'-GAACAACATCTCTGGTATTGAAGA-3'
Ppia	5'-ATTATGGCGTGTAAAGTCACCA-3'
(housekeeping)	5'-AGCACTGGAGAGAAAGGATT-3'
